# Association Between Mutations in DNA Damage Response Genes and Efficacy of Immune Checkpoint Inhibitor Therapy in Non-Small Cell Lung Cancer: A Systematic Review and Meta-Analysis

**DOI:** 10.3390/cancers18162553

**Published:** 2026-08-09

**Authors:** Huixia Li, Wing San Yiu, Guo-Min Li, Feng-Ming (Spring) Kong

**Affiliations:** 1 Department of Clinical Oncology, Li Ka Shing Faculty of Medicine, The University of Hong Kong, Hong Kong SAR, China; lihuixia@connect.hku.hk (H.L.); yiuws@connect.hku.hk (W.S.Y.); 2 Department of Clinical Oncology, The University of Hong Kong–Shenzhen Hospital, Shenzhen 518053, China; 3 Chinese Institute for Cancer Research, Chinese Institutes for Medical Research, Beijing 100069, China; gmli@cimrbj.ac.cn

**Keywords:** non-small cell lung cancer, DNA damage response, immune checkpoint inhibitors, meta-analysis, predictive biomarker

## Abstract

Background: Immunotherapy has improved outcomes in non-small cell lung cancer (NSCLC), but reliable predictive biomarkers remain limited. DNA damage response (DDR) gene mutations may enhance tumor immunogenicity and response to immune checkpoint inhibitors (ICIs), yet clinical findings remain inconsistent. We aimed to systematically evaluate the association between DDR gene mutations and ICI outcomes in NSCLC. Methods: PubMed and Embase were searched for eligible studies. Data on objective response rate (ORR), progression-free survival (PFS), and overall survival (OS) were extracted and pooled using random-effects models. Subgroup analyses were performed by treatment regimen. Results: A total of 13 studies involving 5989 patients were included from 606 records. The pooled RR for ORR comparing DDR wild-type with DDR-mutated patients was 0.63 (95% CI: 0.49–0.82, *p* < 0.001), indicating higher ORR in the DDR-mutated group. DDR mutations were also associated with improved PFS (HR = 0.56, 95% CI: 0.43–0.74, *p* < 0.001) and OS (HR = 0.82, 95% CI: 0.68–0.98, *p* < 0.05). In subgroup analyses, benefits were more consistent with ICI monotherapy, including improved PFS (HR = 0.59, 95% CI: 0.44–0.79, *p* < 0.001) and ORR (RR = 0.60, 95% CI: 0.44–0.82, *p* = 0.001), while OS showed a nonsignificant trend. Evidence in the ICI-plus-chemotherapy subgroup remained inconclusive because of the limited number of available studies and substantial between-study heterogeneity. Conclusions: DDR gene mutations were associated with improved clinical outcomes in NSCLC patients treated with ICIs, particularly in the ICI monotherapy setting.

## Simple Summary

Non-small cell lung cancer is one of the most common causes of cancer death worldwide. Immunotherapy works by helping the immune system detect and attack cancer cells. Although immunotherapy has transformed the treatment of NSCLC, only a subset of patients achieves durable clinical benefit, and reliable biomarkers for patient selection remain limited. When the genes responsible for repairing damaged DNA are mutated, tumors may become more detectable to the immune system. We conducted the first meta-analysis on this topic, combining data from 13 studies including 5989 patients with non-small cell lung cancer. Patients carrying DNA repair gene mutations had higher response rates and lived longer after immunotherapy compared to those without such mutations. This pattern was more consistent among patients who received immunotherapy alone, whereas evidence for immunotherapy combined with chemotherapy remains limited and inconclusive. Our findings support further investigation of DNA repair gene mutations as candidate biomarkers of immunotherapy outcomes.

## 1. Introduction

Non-small cell lung cancer (NSCLC) is a leading cause of cancer-related mortality worldwide [1]. Although immune checkpoint inhibitors (ICIs), such as pembrolizumab and nivolumab, have improved treatment outcomes, only a subset of patients derives durable clinical benefit [2]. For unselected NSCLC patients, the objective response rate (ORR) with ICI monotherapy is historically limited to approximately 20% [3]. Currently available biomarkers, such as programmed death-ligand 1 (PD-L1) expression and tumor mutational burden (TMB), are limited by intertumoral heterogeneity [4], dynamic expression [3,5], and technical challenges [6]. Therefore, more robust biomarkers are needed to improve patient selection for immunotherapy in NSCLC.

DNA damage response (DDR) gene mutations have emerged as a promising class of candidate biomarkers for immunotherapy efficacy [7]. DDR pathways are essential for maintaining genomic stability by correcting DNA lesions caused by endogenous and exogenous insults [8]. The DDR system comprises multiple pathways to maintain genomic integrity, such as direct repair (DR), base excision repair (BER), mismatch repair (MMR), nucleotide excision repair (NER), homologous recombination (HR), and nonhomologous end joining (NHEJ) [9]. Defects in DDR genes lead to genomic instability, accumulation of somatic mutations, and release of cytosolic double-stranded DNA fragments [10]. These cytosolic DNA fragments activate the cyclic GMP-AMP synthase (cGAS)–stimulator of interferon genes (STING) pathway [11], inducing type I interferon signaling and enhancing antigen presentation, dendritic cell activation, and CD8^+^ T-cell infiltration [12]. Consequently, DDR deficiency can remodel the tumor microenvironment into a T cell-inflamed phenotype, thereby sensitizing tumors to immune checkpoint blockade.

Accumulating clinical evidence suggests that DDR gene mutations may serve as predictive biomarkers for ICI efficacy across cancer types [13,14,15,16]. Given the biological importance of DNA repair in shaping tumor immunogenicity, DDR gene mutations represent a particularly relevant biomarker candidate in NSCLC. In NSCLC, several retrospective studies have explored the association between DDR gene mutations and clinical outcomes following ICI therapy, with several reporting favorable associations with response or survival [17,18,19,20,21,22,23,24,25,26,27,28,29]. DDR status does not represent a single molecular entity. Across the included studies, tumors were classified using different gene panels, repair pathways, and variant criteria. A pooled analysis can nevertheless address whether an association is observed across independent cohorts despite these differences. It also allows the direction of the association to be compared across response and survival outcomes and helps identify where the evidence is consistent and where uncertainty remains. We therefore examined the overall association between study-defined DDR alterations and ICI outcomes and then assessed the results by treatment regimen and through sensitivity analyses. This approach was intended to summarize the available clinical evidence without assuming that all DDR genes or pathways have equivalent effects. However, no NSCLC-specific quantitative synthesis has comprehensively integrated response and survival outcomes or evaluated the available evidence within distinct treatment contexts. Therefore, we conducted a systematic review and meta-analysis to provide pooled estimates across ORR, PFS, and OS, assess the consistency and robustness of the reported associations, and evaluate the evidence separately for ICI monotherapy and ICI-based combination therapy.

## 2. Methods

### 2.1. Literature Search and Selection Criteria

PubMed and Embase were searched using controlled vocabulary and free-text terms related to non-small cell lung cancer, immune checkpoint inhibitors, and DNA damage response and repair. Reference lists of eligible studies and relevant reviews were manually screened to identify additional studies. Controlled vocabulary terms, including MeSH terms in PubMed and Emtree terms in Embase, were combined with relevant free-text terms. The full electronic search strategies for each database are provided in Section S1. Eligible studies met the following criteria: (1) observational cohort studies enrolling patients with advanced, unresectable, recurrent, or metastatic NSCLC treated with ICIs; (2) studies reporting comparative outcomes according to DDR alteration status; (3) studies evaluating ICI monotherapy or ICI-based combination therapy; (4) studies reporting available data on OS, PFS, or ORR; (5) studies published in English. When a source publication included a broader range of disease stages or reported the stage at initial diagnosis, only outcome data from the eligible ICI-treated analytic cohort were extracted.

### 2.2. Data Extraction and Quality Assessment

The data extracted from the selected studies included general characteristics such as first author, publication year, journal, study design, total sample size, number of DDR-altered and DDR-wild-type patients, stage, DDR-altered genes, immunotherapy types and treatment duration. When a publication reported more than one eligible analytic cohort, data from each cohort were extracted separately according to the DDR definition and treatment regimen. For OS and PFS, the reported HRs and corresponding 95% CIs were recorded. For ORR, comparative response data for the DDR-altered and DDR-wild-type groups were extracted. For source publications that included broader disease-stage populations or reported stage at initial diagnosis, we recorded the disease stage of the eligible ICI-treated analytic cohort from which the outcome data were extracted.

This review was reported in accordance with the Preferred Reporting Items for Systematic Reviews and Meta-Analyses (PRISMA) 2020 statement. The completed PRISMA 2020 checklist is provided in the Supplementary Materials. This review was not prospectively registered in a public registry (e.g., PROSPERO). All study data were extracted independently by two readers (Li, Yiu) and the quality of the included studies was assessed by two investigators independently using the Newcastle–Ottawa Scale for cohort studies (Table S1). Disagreements in the quality assessment were resolved by discussion.

Because no universally accepted clinical definition of DDR alteration currently exists in NSCLC immunotherapy studies, DDR status was extracted according to the definitions used in the original studies rather than reclassified by the review authors. These definitions included broad DDR panels, pathway-specific alterations, and selected single-gene or gene-subset analyses. To improve biological consistency, genes or pathways classified as DDR-related in the included studies were cross-checked against the curated Human DNA Repair Genes resource maintained by the Wood Laboratory at UT MD Anderson. Studies were eligible when the reported DDR-related genes were covered by this reference list, whose table information was last updated in November 2025.

### 2.3. Statistical Analysis

Meta-analyses were conducted in accordance with Cochrane guidelines using Review Manager (RevMan) version 5.4. Statistical heterogeneity was assessed using the χ^2^ test and quantified with the I^2^ statistic, with *p* < 0.10 considered indicative of significant heterogeneity and I^2^ > 50% denoting substantial heterogeneity. A random-effects model was applied to calculate pooled hazard ratios (HRs) and risk ratios (RRs). Meta-analysis results for overall survival (OS) and progression-free survival (PFS) were expressed as HRs with corresponding 95% confidence intervals (CIs), and objective response rate (ORR) results were reported as RRs with 95% CIs. All *p*-values were two-sided, and *p* < 0.05 was considered statistically significant. Funnel plots were used to visually assess potential publication bias. Leave-one-out sensitivity analyses were conducted by sequentially excluding each study and recalculating the pooled estimate and heterogeneity. These analyses were limited to outcomes with enough contributing studies and were not performed for the ICI-plus-chemotherapy subgroup, which included only three studies. Funnel plots were interpreted descriptively, given the limited number of studies available for several outcomes.

## 3. Results

### 3.1. Study Selection

**Figure 1:**
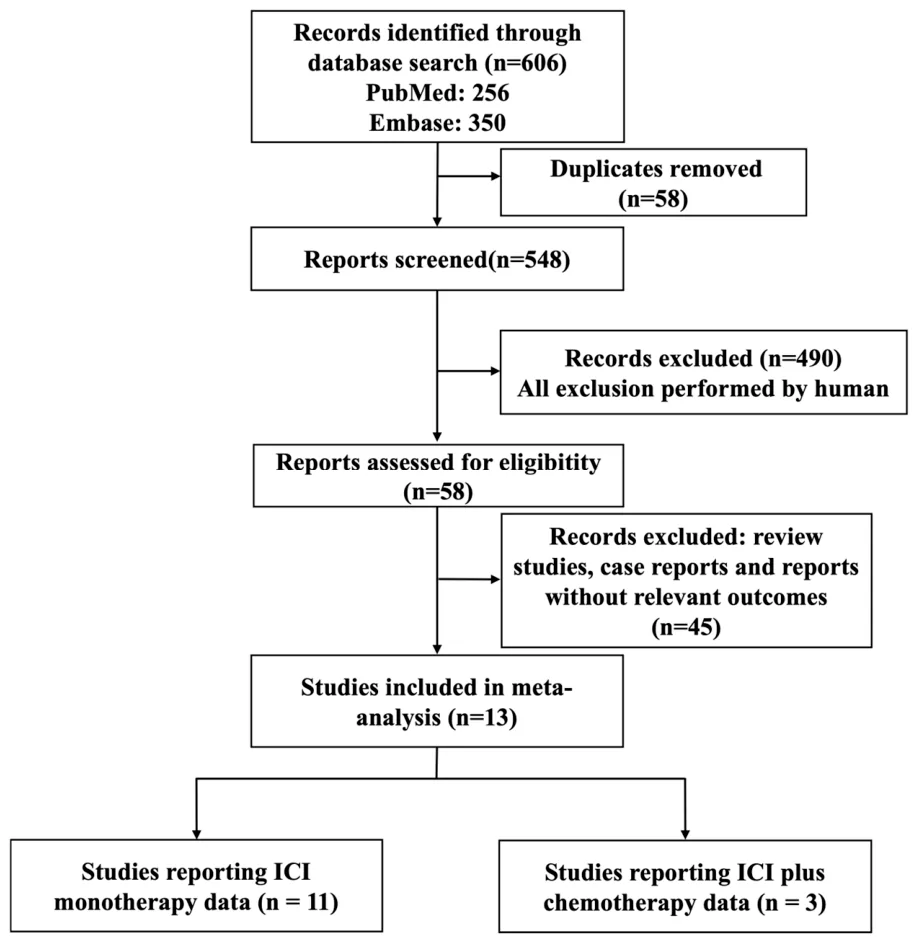
PRISMA flow diagram of the study selection process. Among the 13 included studies, 11 reported outcomes for ICI monotherapy and 3 reported outcomes for ICIs plus chemotherapy. One study contributed data to both treatment subgroups.

The literature search identified 606 records from PubMed and Embase. After removal of 58 duplicate records, 548 records remained for title and abstract screening. During the initial screening, 490 records were excluded because they were unrelated to the study topic based on their titles and abstracts. The full texts of the remaining 58 articles were assessed for eligibility. Of these, 45 were excluded because they were reviews or case reports, used ineligible study designs, involved inappropriate patient populations, or did not report outcomes of interest. As shown in Figure 1, 13 studies [17,18,19,20,21,22,23,24,25,26,27,28,29] were included in this meta-analysis. Of the included studies, 10 provided OS data (*n* = 5363), 13 reported PFS data (*n* = 5033), and 11 reported ORR data (*n* = 3477). The basic characteristics of the included studies are summarized in Table 1.

The included studies covered both immunotherapy monotherapy and combination regimens. However, we only identified studies involving immunotherapy alone or immunotherapy combined with chemotherapy. No eligible studies involving immunotherapy plus radiotherapy or immunotherapy plus targeted therapy in DDR-altered NSCLC were found. Of the 3 studies involving immunotherapy plus chemotherapy, only one provided OS data.

### 3.2. Overall Impact of DDR Gene Mutations on Immunotherapy Efficacy in NSCLC

**Figure 2:**
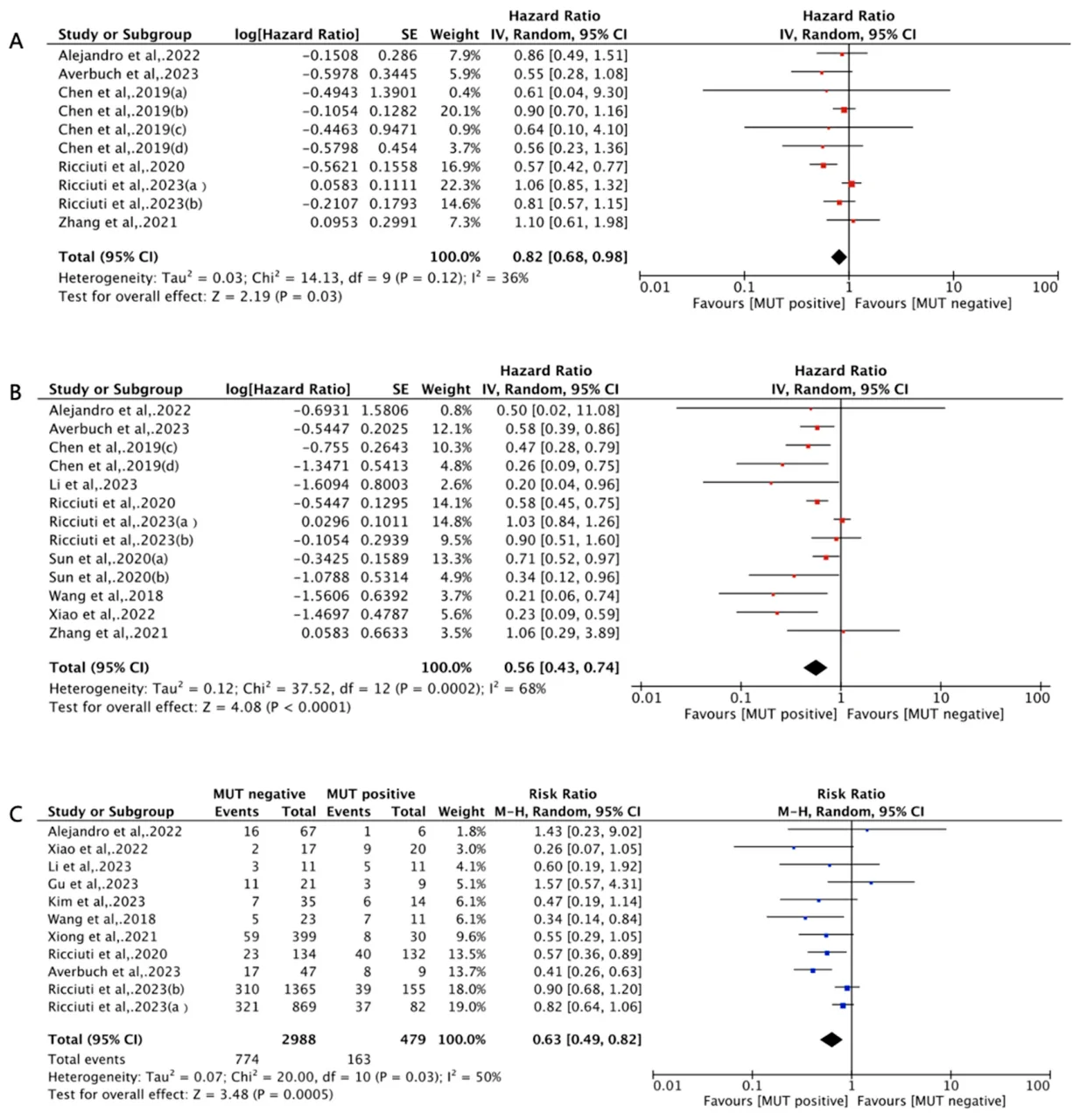
Overall association between DDR alteration status and ICI outcomes in patients with NSCLC. (**A**) Pooled hazard ratio (HR) for overall survival; (**B**) pooled HR for progression-free survival; and (**C**) pooled risk ratio (RR) for objective response rate. HRs were calculated for DDR-altered versus DDR-wild-type tumors, whereas the RR for objective response was calculated for DDR-wild-type versus DDR-altered tumors; values below 1 therefore favor the DDR-altered group [17,18,19,20,21,22,23,24,25,26,27,28,29].

Compared with DDR-wild-type patients, patients with DDR alterations had significantly better clinical outcomes following ICI treatment. ORR was higher in the DDR-altered group (RR for DDR-wild-type versus DDR-altered tumors = 0.63, 95% CI: 0.49–0.82, *p* = 0.0005), with moderate heterogeneity (I^2^ = 50%, χ^2^ = 20.00, *p* = 0.03; τ^2^ = 0.07). The pooled HR for OS was 0.82 (95% CI: 0.68–0.98, *p* = 0.03), with low-to-moderate heterogeneity (I^2^ = 36%, χ^2^ = 14.13, *p* = 0.12; τ^2^ = 0.03). PFS was also longer in the DDR-altered group (HR = 0.56, 95% CI: 0.43–0.74, *p* < 0.0001), with substantial heterogeneity (I^2^ = 68%, χ^2^ = 37.52, *p* = 0.0002; τ^2^ = 0.12) (Figure 2A–C). These findings indicate an association between DDR alteration status and improved ICI outcomes in NSCLC, although the retrospective nature of the evidence and the lack of uniform non-ICI comparators limit definitive inference regarding predictive value.

To evaluate the robustness of the pooled estimates, a sensitivity analysis was performed by sequentially excluding individual studies. One study (Ricciuti, 2023[a]) contributed notably to heterogeneity in the PFS analysis, likely reflecting its ATM-specific biomarker definition and differences in cohort composition. After excluding this study, heterogeneity decreased from 68% to 34%, while the pooled HR remained stable (from 0.56 [95% CI, 0.43–0.74] to 0.54 [95% CI, 0.43–0.68]), indicating that the overall findings were robust (Figure S1).

Publication bias was assessed through visual inspection of funnel plots for each primary outcome. In the overall population, funnel plots for ORR, PFS, and OS (Figure S2A–C) appeared approximately symmetric, suggesting no substantial evidence of publication bias.

### 3.3. Subgroup Analyses by Treatment Regimen

#### 3.3.1. ICI Monotherapy

**Figure 3:**
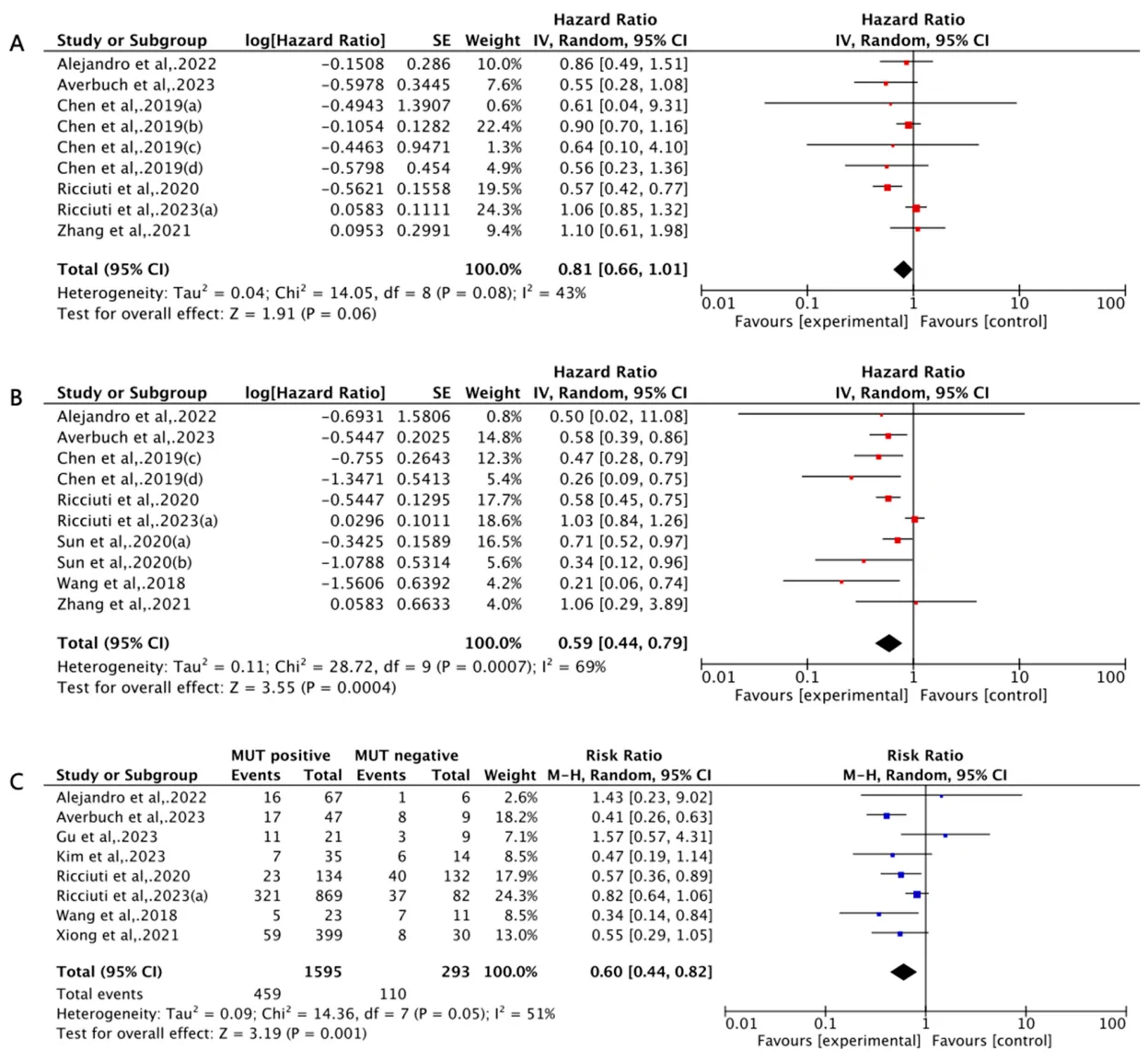
Association between DDR alteration status and clinical outcomes in patients with NSCLC receiving ICI monotherapy. (**A**) Pooled HR for overall survival; (**B**) pooled HR for progression-free survival; and (**C**) pooled RR for objective response rate. HRs were calculated for DDR-altered versus DDR-wild-type tumors, whereas the RR for objective response was calculated for DDR-wild-type versus DDR-altered tumors; values below 1 therefore favor the DDR-altered group [17,18,19,20,21,23,24,25,26,28,29].

To explore whether treatment regimen influenced the observed associations, we performed subgroup analyses according to ICI monotherapy versus chemo-immunotherapy. The signal appeared more consistent in the monotherapy subgroup, whereas evidence in the chemo-immunotherapy subgroup remained limited. In the subgroup of patients receiving ICIs as monotherapy, those with DDR-altered tumors showed a trend toward improved OS compared to DDR wild-type patients, although the difference did not reach statistical significance (HR = 0.81, 95% CI: 0.66–1.01, *p* = 0.06). Heterogeneity was moderate (I^2^ = 43%, χ^2^ = 14.05, *p* = 0.08; τ^2^ = 0.04), and a random-effects model was applied. For PFS, patients with DDR alterations experienced significantly longer survival (HR = 0.59, 95% CI: 0.44–0.79, *p* < 0.001), with substantial heterogeneity across studies (I^2^ = 69%, χ^2^ = 28.72, *p* = 0.0007; τ^2^ = 0.11). The ORR was also significantly higher in the DDR-altered group (RR = 0.60, 95% CI: 0.44–0.82, *p* = 0.001), with moderate heterogeneity (I^2^ = 51%, χ^2^ = 14.36, *p* = 0.05; τ^2^ = 0.09) (Figure 3A–C).

Sensitivity analyses were performed to assess the robustness of the pooled estimates for ORR, PFS, and OS. One study by Ricciuti et al. [18] consistently contributed to elevated heterogeneity across all three outcomes, with notably higher effect sizes and wide confidence intervals. After excluding this study, heterogeneity decreased substantially from 43% to 15% for OS, 69% to 9% for PFS, and 51% to 30% for ORR, while the pooled effect estimates remained stable. Specifically, the pooled HR for OS changed from 0.81 (95% CI: 0.66–1.01) to 0.75 (95% CI: 0.61–0.92); the pooled HR for PFS from 0.59 (95% CI: 0.44–0.79) to 0.57 (95% CI: 0.48–0.68); and the pooled RR for ORR from 0.60 (95% CI: 0.44–0.82) to 0.54 (95% CI: 0.39–0.74) (Figure S3A–C). Notably, the pooled HR for OS became statistically significant after excluding this study, suggesting that the survival benefit may have been previously masked by study-level variability. Furthermore, visual inspection of funnel plots for this monotherapy subgroup (Figure S4A–C) revealed a generally symmetrical distribution, suggesting that the results for this specific regimen were unlikely to be substantially affected by publication bias. Overall, the association between DDR gene mutations and favorable clinical outcomes appeared more consistent in the ICI monotherapy subgroup.

#### 3.3.2. ICIs Plus Chemotherapy

**Figure 4:**
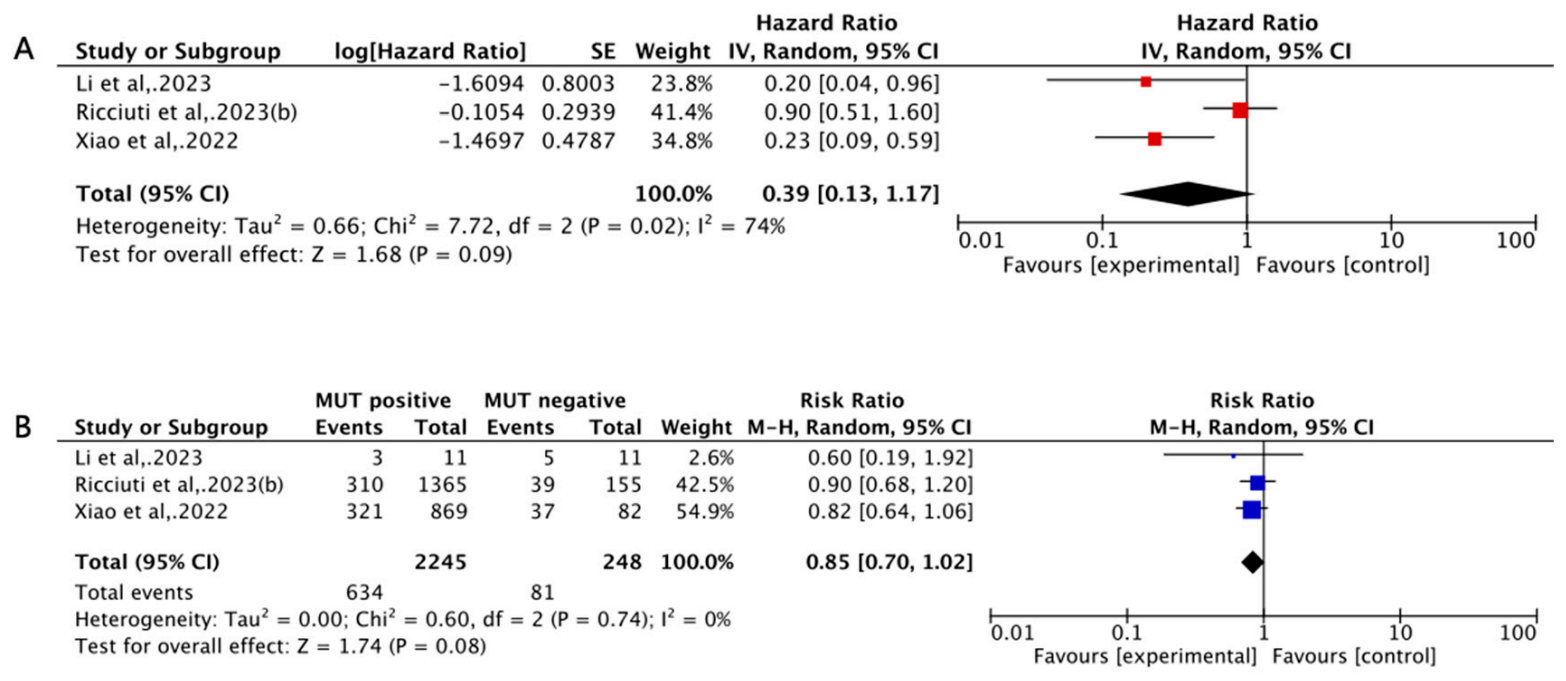
Association between DDR alteration status and clinical outcomes in patients with NSCLC receiving ICIs plus chemotherapy. (**A**) Pooled HR for progression-free survival and (**B**) pooled RR for objective response rate. The HR was calculated for DDR-altered versus DDR-wild-type tumors, whereas the RR was calculated for DDR-wild-type versus DDR-altered tumors; values below 1 therefore favor the DDR-altered group [18,22,27].

In the subgroup of patients receiving ICIs in combination with chemotherapy, PFS appeared longer in the DDR-altered group than in the DDR-wild-type group, although the difference did not reach statistical significance (HR = 0.39, 95% CI: 0.13–1.17, *p* = 0.09). Substantial heterogeneity was observed (I^2^ = 74%, χ^2^ = 7.72, *p* = 0.02; τ^2^ = 0.66), indicating notable between-study variability. ORR did not differ significantly between the DDR-altered and DDR-wild-type groups (RR = 0.85, 95% CI: 0.70–1.02, *p* = 0.08), with no substantial heterogeneity detected (I^2^ = 0%, χ^2^ = 0.60, *p* = 0.74; τ^2^ = 0.00) (Figure 4A,B). Only one study reported OS data for patients treated with ICIs plus chemotherapy; therefore, no pooled OS analysis was conducted for this subgroup.

Because only three studies were included in this subgroup, sensitivity analysis and publication bias assessment were not performed, as these analyses are unreliable with such a small number of studies.

## 4. Discussion

This meta-analysis of 13 studies involving 5989 patients showed that DDR alterations were associated with a higher ORR (RR for DDR-wild-type versus DDR-altered tumors = 0.63), longer PFS (HR = 0.56), and longer OS (HR = 0.82) among patients with NSCLC treated with ICIs. In regimen-specific analyses, the associations with PFS and ORR were more consistent in the ICI monotherapy subgroup, whereas evidence for ICI-based combination therapy remained inconclusive. Leave-one-out sensitivity analyses showed that the direction of the pooled estimates remained stable after individual studies were excluded, although heterogeneity varied across analyses.

To our knowledge, this is the first NSCLC-specific systematic review and meta-analysis to quantitatively synthesize evidence on the association between DDR alterations and clinical outcomes following ICI treatment, including ORR, PFS, and OS. Previous retrospective studies have explored the prognostic and predictive relevance of DDR-related alterations in this setting and have generally suggested a potential association with immunotherapy benefit; however, their findings have been inconsistent. The contribution of the present study extends beyond increasing the aggregated sample size. By integrating evidence from independent cohorts, this meta-analysis provides pooled effect estimates, evaluates the consistency of the associations across response and survival outcomes, assesses their robustness through sensitivity analyses, and examines the available evidence within distinct treatment settings. In the ICI monotherapy subgroup, DDR alterations were associated with improved PFS and ORR, indicating a relatively consistent association in this setting. In contrast, the predictive relevance of DDR alterations in ICI-based combination therapy remains inconclusive. The PFS analysis included only three studies and showed substantial between-study heterogeneity, while only one study reported eligible OS data. Therefore, the absence of a statistically significant association should not be interpreted as evidence that DDR alterations lack predictive relevance in combination therapy. Interpretation in this setting is also complicated by the independent therapeutic effects of chemotherapy, which may influence tumor burden, immune activity, and survival outcomes and make the specific contribution of DDR status to ICI sensitivity difficult to isolate. The currently available evidence is insufficient to determine whether treatment regimen modifies the association between DDR alterations and ICI outcomes. Although these regimen-specific findings should be considered hypothesis-generating, the subgroup analysis remains informative because it identifies the treatment setting in which the current evidence is more consistent and highlights an important evidence gap in combination therapy. Future prospective studies should evaluate DDR alterations within clearly defined treatment regimens, apply standardized DDR definitions, and include sufficient sample sizes to permit formal treatment-by-biomarker interaction analyses.

**Figure 5:**
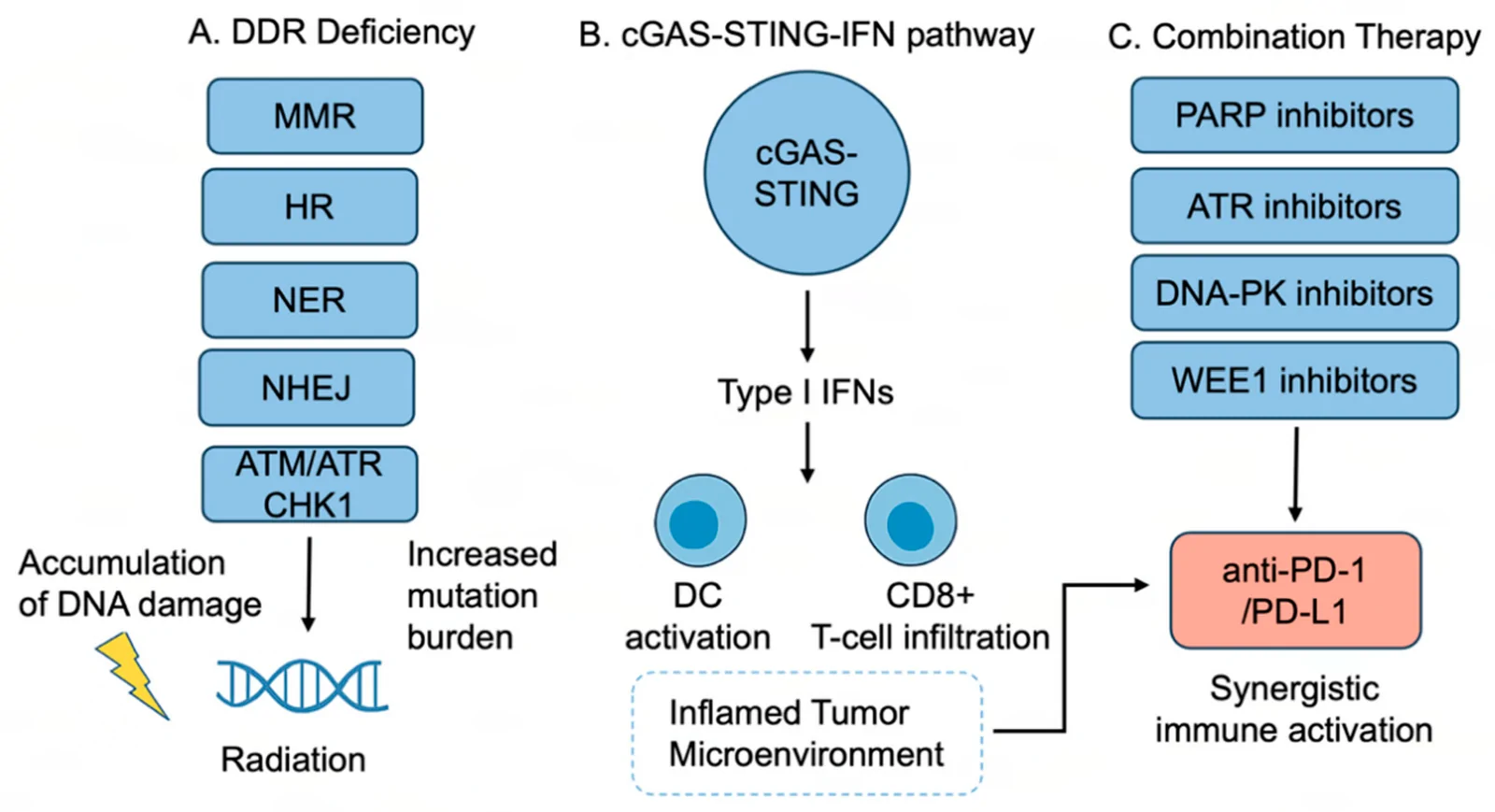
Biological rationale linking DDR deficiency to immune activation and potential therapeutic combinations. (**A**) Defects in mismatch repair (MMR), homologous recombination (HR), nucleotide excision repair (NER), nonhomologous end joining (NHEJ), or ATM/ATR/CHK1 signaling may lead to the accumulation of unrepaired DNA and an increased mutation burden; radiation represents an additional source of DNA damage. (**B**) DNA damage may activate cGAS–STING/type I interferon signaling, thereby promoting dendritic cell activation, CD8^+^ T-cell infiltration, and an inflamed tumor microenvironment. (**C**) Selected DDR-targeting agents, including PARP, ATR, DNA-PK, and WEE1 inhibitors, may enhance antitumor immune activation when combined with PD-1/PD-L1 blockade.

The observed associations are biologically plausible because DDR defects may increase mutational burden and neoantigen generation and activate innate immune signaling, including the cGAS–STING/type I interferon pathway [30]. These effects may promote an inflamed tumor microenvironment and facilitate responses to immune checkpoint blockade. The same biological rationale has prompted investigation of strategies that increase unrepaired DNA damage or inhibit selected DDR pathways in combination with ICIs. Their activity is likely to depend on the molecular target, tumor genotype, treatment schedule, and clinical setting. Figure 5 summarizes these proposed relationships.

From a clinical and translational perspective, the present findings support further investigation of DDR alterations as candidate biomarkers of immunotherapy benefit, particularly within defined treatment contexts. One important implication is that DDR should not be regarded as a biologically uniform biomarker class. The studies included in this meta-analysis differed substantially in the genes assessed, the pathways represented, the variant types included, and the criteria used to define DDR positivity. These differences are not merely methodological; they likely reflect meaningful biological heterogeneity [31] and should therefore be considered when interpreting the pooled estimates. Within this context, the present meta-analysis provides a quantitative summary of the overall association across study-defined DDR categories without assuming identical effects for every DDR gene or pathway. Alterations affecting mismatch repair, homologous recombination, or checkpoint signaling may have distinct mechanistic consequences, highlighting the value of pathway-resolved analyses for refining biomarker interpretation [32]. Beyond these pathway-specific differences, a binary classification of DDR-altered versus DDR-wild-type tumors may not fully capture the potential relevance of the number and co-mutation pattern of DDR alterations. Wang et al. and Xiong et al. suggested that co-mutations involving multiple DDR pathways may represent potential biomarkers of ICI benefit [26,28]. Ricciuti et al. further reported a stepwise increase in median TMB among tumors with no deleterious DDR mutation, one DDR mutation, and two or more DDR mutations (7.6, 10.6, and 15.2 mutations/Mb, respectively; *p* < 0.001) [19]. This observation suggests that the cumulative burden of DDR alterations may be associated with increasing genomic instability and may partly contribute to the heterogeneity observed across studies. However, a higher TMB does not by itself establish greater clinical benefit from ICIs, and the predictive relevance of multiple DDR alterations requires validation in larger prospective cohorts.

Gene-specific effects and differences in variant-pathogenicity classification may represent additional sources of heterogeneity. Ricciuti et al. reported no significant differences in ORR, PFS, or OS between patients with deleterious ATM mutations and those with ATM-wild-type tumors following either PD-(L)1 monotherapy or platinum-based chemo-immunotherapy [18]. In that study, loss-of-function ATM alterations and missense variants predicted to be pathogenic were classified as ATM-mutant, whereas missense variants not predicted to be pathogenic were considered benign and included in the ATM-wild-type group. This finding illustrates how variant-classification criteria may affect the composition of biomarker-defined groups and potentially contribute to between-study heterogeneity. Standardized pathogenicity criteria and additional functional validation will therefore be important in future studies of DDR alterations. Future research should therefore move beyond broad binary DDR classification toward pathway-resolved models that can identify which specific alterations are clinically informative and under which therapeutic conditions. Such an approach may refine patient selection, improve biological interpretability, and support the development of biomarker-enriched trials involving ICI monotherapy or rational combinations with DDR-targeting agents, DNA damage-inducing modalities, or radiotherapy. Emerging approaches involving PARP inhibitors [33], ATR inhibitors [34], DNA-PK inhibitors, WEE1 inhibitors, and radiotherapy-based combinations are of particular interest in this context [35].

Several considerations should be noted when interpreting these findings. First, the number of available studies was limited, particularly for regimen-specific subgroup analyses. Nevertheless, by integrating 13 studies involving 5989 patients, the present study represents, to our knowledge, the first NSCLC-specific systematic review and meta-analysis to quantitatively evaluate the association between DDR alterations and ICI outcomes and provides an important foundation for future prospective validation. Second, most studies were retrospective, and residual confounding could not be fully excluded; nevertheless, the pooled analysis provides hypothesis-generating evidence supporting further evaluation of DDR alterations as candidate biomarkers in this setting. Third, DDR status was defined according to the criteria used in each original study, resulting in variation in the genes and pathways assessed, the variant types included, and the definitions of DDR positivity. Accordingly, the pooled estimates represent the average association across study-defined DDR categories; nevertheless, they provide a quantitative synthesis of the currently available evidence and a basis for future standardized, pathway-specific studies. Fourth, key covariates, including PD-L1 expression, TMB, smoking history, and STK11/KEAP1 co-mutations, were not consistently available [36,37], limiting assessment of the independent predictive value of DDR status beyond established biomarkers. Prospective studies using standardized DDR definitions, prespecified biomarker assessments, and integrated multivariable and pathway-level models are needed to clarify the clinical utility of DDR alterations in biomarker-guided immunotherapy for NSCLC.

## 5. Conclusions

DDR alterations were associated with improved outcomes in patients with NSCLC treated with ICIs, with more consistent associations in the ICI monotherapy subgroup. Evidence for ICIs plus chemotherapy remains inconclusive because few studies were available and between-study heterogeneity was substantial. Prospective studies using standardized, pathway-resolved definitions are needed to determine which DDR alterations may be clinically informative in specific treatment settings.

## Figures and Tables

**Table 1:** Studies included in the meta-analysis.

First Author, Year of Publication	Country/Nation	Study Design	DDR Genes/Definitions	Number of Patients	Number of DDR Mutation	Number of DDR Wild Type	Treatment	Study Population/Disease Setting	Outcomes
Chen, 2019 [17]	multicountry	Retrospective cohort study	MMR	350	17	333	ICI monotherapy	Advanced NSCLC	OS
		Retrospective cohort study	TP53/ATM	429	22	407	ICI monotherapy	Advanced NSCLC	OS, PFS
		Retrospective cohort study	MMR gene	429	33	392	ICI monotherapy	Advanced NSCLC	OS, PFS
		Retrospective cohort study	POLE/D1	429	17	412	ICI monotherapy	Advanced NSCLC	OS, PFS
Ricciuti, 2023 [18]	USA	Retrospective cohort study	ATM	1522	155	1367	PD-(L)1 monotherapy	Advanced NSCLC	OS, PFS, ORR
		Retrospective cohort study	ATM	951	82	869	chemo-immunotherapy	Advanced/metastatic NSCLC	OS, PFS, ORR
Ricciuti, 2020 [19]	USA	Retrospective cohort study	DDR (53-gene panel)	266	132	134	PD-(L)1 therapy	Advanced NSCLC	OS, PFS, ORR
Sun, 2020 [20]	multicountry	Retrospective cohort study	TP53	147	85	73	ICI monotherapy	Advanced NSCLC	PFS
		Retrospective cohort study	TP53	59	27	32	ICI monotherapy	Advanced NSCLC	PFS
Zhang, 2021 [21]	China	Retrospective cohort study	PALB2	429	NA	NA	Immunotherapy monotherapy	Advanced NSCLC	OS
		Retrospective cohort study	PALB2	240	NA	NA	Immunotherapy monotherapy	Advanced NSCLC	PFS
Xiao, 2022 [22]	China	Retrospective cohort study	DDR (35-gene panel)	37	20	17	chemo-immunotherapy	Advanced NSCLC	PFS, ORR
Averbuch, 2023 [23]	Israel	Retrospective cohort study	DDR (30-gene panel)	56	9	47	Immunotherapy monotherapy	Stage IVNSCLC	OS, ORR
Gu, 2023 [24]	China	Retrospective cohort study	DDR (seven pathways)	32	11	21	Immunotherapy monotherapy	Stage III-IV NSCLC	ORR
Kim, 2023 [25]	Korean	Retrospective cohort study	DDR (54-gene panel)	55	14	41	Immunotherapy and chemo-immunotherapy	Advanced NSCLC	ORR
Xiong, 2021 [26]	multicountry	Retrospective cohort study	DDR (seven pathways)	429	30	399	ICI monotherapy	Advanced NSCLC	ORR
Li, 2023 [27]	China	Retrospective cohort study	HRD	22	11	11	chemo-immunotherapy	Metastatic NSCLC	PFS, ORR
Wang, 2018 [28]	multicountry	Retrospective cohort study	HRR–BER/HRR–MMR co-mutation	34	11	23	ICI monotherapy	Advanced NSCLC	ORR
Alejandro, 2022 [29]	Spain	Retrospective cohort study	MMR	73	6	67	ICI monotherapy	Advanced/metastatic NSCLC	OS, PFS, ORR

## Data Availability

No new data were created or analyzed in this study. All data analyzed in this meta-analysis were obtained from the published studies cited in the article.

## Supplementary Materials

The following supporting information can be downloaded at https://www.mdpi.com/article/10.3390/cancers18162553/s1. Section S1: Full search strategy; Table S1: Newcastle–Ottawa Scale (NOS) assessment of included studies; Figure S1: Sensitivity analysis for progression-free survival in the overall population; Figure S2: Funnel plots for overall survival, progression-free survival, and objective response rate in the overall population; Figure S3: Sensitivity analyses for overall survival, progression-free survival, and objective response rate in the ICI monotherapy subgroup; Figure S4: Funnel plots for overall survival, progression-free survival, and objective response rate in the ICI monotherapy subgroup; PRISMA 2020 Checklist.
