# Supplementary material for: Association Between Mutations in DNA Damage Response Genes and Efficacy of Immune Checkpoint Inhibitor Therapy in Non-Small Cell Lung Cancer: A Systematic Review and Meta-Analysis
Source: Cancers (Basel). 2026 Aug 9;18(16):2553. doi: 10.3390/cancers18162553 (PMC13510565; doi:10.3390/cancers18162553)
Supplement: Supplementary file 1 [file cancers-18-02553-s001.zip › cancers-4435576-supplementary.pdf]

Supplementary Materials

Association Between Mutations in DNA Damage Response Genes and Efficacy of Immune Checkpoint Inhibitor Therapy in Non-Small Cell Lung Cancer: A Systematic Review and Meta-Analysis

Section S1. Full Search Strategy

PubMed search strategy (combining MeSH terms and free-text terms for lung cancer, immune checkpoint inhibitors, and DNA damage response/repair):

```
((("Lung Neoplasms"[MeSH Terms]) OR (((((((((((((((((Lung Neoplasms[All Fields]) OR (Pulmonary
Neoplasms[All Fields])) OR (Neoplasms, Lung[All Fields])) OR (Lung Neoplasm[All Fields])) OR
(Neoplasm, Lung[All Fields])) OR (Neoplasms, Pulmonary[All Fields])) OR (Neoplasm, Pulmonary[All
Fields])) OR (Pulmonary Neoplasm[All Fields])) OR (Lung Cancer[All Fields])) OR (Cancer, Lung[All
Fields])) OR (Cancers, Lung[All Fields])) OR (Lung Cancers[All Fields])) OR (Pulmonary Cancer[All
Fields])) OR (Cancer, Pulmonary[All Fields])) OR (Cancers, Pulmonary[All Fields])) OR (Pulmonary
Cancers[All Fields])) OR (Cancer of the Lung[All Fields])) OR (Cancer of Lung[All Fields]))) AND
(((("Immunotherapy"[MeSH Terms]) OR ((Immunotherapy[All Fields]) OR (Immunotherapies[All
Fields]))) OR ("Immune Checkpoint Inhibitors"[MeSH Terms])) OR
(((((((((((((((((((((((((((((((((Immune Checkpoint Inhibitors[All Fields]) OR (Checkpoint
Inhibitors, Immune[All Fields])) OR (Immune Checkpoint Inhibitor[All Fields])) OR (Checkpoint
Inhibitor, Immune[All Fields])) OR (Immune Checkpoint Blockers[All Fields])) OR (Checkpoint
Blockers, Immune[All Fields])) OR (Immune Checkpoint Blockade[All Fields])) OR (Checkpoint
Blockade, Immune[All Fields])) OR (Immune Checkpoint Inhibition[All Fields])) OR (Checkpoint
Inhibition, Immune[All Fields])) OR (PD-L1 Inhibitors[All Fields])) OR (PD L1 Inhibitors[All
Fields])) OR (PD-L1 Inhibitor[All Fields])) OR (PD L1 Inhibitor[All Fields])) OR (Programmed
Death-Ligand 1 Inhibitors[All Fields])) OR (Programmed Death Ligand 1 Inhibitors[All Fields])) OR
(PD-1-PD-L1 Blockade[All Fields])) OR (Blockade, PD-1-PD-L1[All Fields])) OR (PD 1 PD L1
Blockade[All Fields])) OR (CTLA-4 Inhibitors[All Fields])) OR (CTLA 4 Inhibitors[All Fields])) OR
(CTLA-4 Inhibitor[All Fields])) OR (CTLA 4 Inhibitor[All Fields])) OR (Cytotoxic T-Lymphocyte-
Associated Protein 4 Inhibitors[All Fields])) OR (Cytotoxic T Lymphocyte Associated Protein 4
Inhibitors[All Fields])) OR (Cytotoxic T-Lymphocyte-Associated Protein 4 Inhibitor[All Fields]))
OR (Cytotoxic T Lymphocyte Associated Protein 4 Inhibitor[All Fields])) OR (PD-1 Inhibitors[All
Fields])) OR (PD 1 Inhibitors[All Fields])) OR (PD-1 Inhibitor[All Fields])) OR (Inhibitor, PD-
1[All Fields])) OR (PD 1 Inhibitor[All Fields])) OR (Programmed Cell Death Protein 1
Inhibitor[All Fields])) OR (Programmed Cell Death Protein 1 Inhibitors[All Fields])))) AND
((((((((("DNA Repair"[MeSH Terms]) OR ((DNA repair[All Fields]) OR (DNA Damage Response[All
Fields]))) OR ("Excision Repair"[MeSH Terms])) OR (((((((((Excision Repair[All Fields]) OR
(Nucleotide Excision Repair[All Fields])) OR (Excision Repair, Nucleotide[All Fields])) OR
(Transcription Coupled Excision Repair[All Fields])) OR (Transcription-Coupled Excision
Repair[All Fields])) OR (Excision Repair, Transcription-Coupled[All Fields])) OR (Transcription-
Coupled Repair[All Fields])) OR (Transcription Coupled Repair[All Fields])) OR (Base Excision
Repair[All Fields])) OR (Excision Repair, Base[All Fields]))) OR ("DNA Mismatch Repair"[MeSH
Terms])) OR ((((mismatch repair[All Fields]) OR (Mismatch Repair, DNA[All Fields])) OR (Repair,
DNA Mismatch[All Fields])) OR (Mismatch Repair[All Fields])) OR (Repair, Mismatch[All Fields])))
OR ("Recombinational DNA Repair"[MeSH Terms])) OR ((((((((((((((((((Recombinational DNA
Repair[All Fields]) OR (DNA Repair, Recombinational[All Fields])) OR (Repair, Recombinational
DNA[All Fields])) OR (Recombinational Repair of DNA[All Fields])) OR (DNA Recombinational
Repair[All Fields])) OR (Homologous Recombinational Repair[All Fields])) OR (Homologous
Recombinational Repairs[All Fields])) OR (Recombinational Repair, Homologous[All Fields])) OR
(Repair, Homologous Recombinational[All Fields])) OR (Homologous Recombination Repair[All
Fields])) OR (Recombination Repair, Homologous[All Fields])) OR (Homologous Recombination Repair
of DNA[All Fields])) OR (Homologous Recombination DNA Repair[All Fields])) OR (Recombination
Repair[All Fields])) OR (Repair, Recombination[All Fields])) OR (Homologous Recombination Double-
Stranded Break DNA Repair[All Fields])) OR (Homologous Recombination Double Stranded Break DNA
Repair[All Fields])) OR (Homology-Directed dsDNA Break Repair[All Fields])) OR (Homology Directed
dsDNA Break Repair[All Fields]))) OR ("DNA End-Joining Repair"[MeSH Terms])) OR
((((((((((((((((((((((DNA End-Joining Repair[All Fields]) OR (DNA End Joining Repair[All
Fields])) OR (End-Joining Repair, DNA[All Fields])) OR (Repair, DNA End-Joining[All Fields])) OR
(End-Joining DNA Repair[All Fields])) OR (DNA Repair, End-Joining[All Fields])) OR (End Joining
DNA Repair[All Fields])) OR (Repair, End-Joining DNA[All Fields])) OR (Microhomology-Mediated End
Joining Repair[All Fields])) OR (Microhomology Mediated End Joining Repair[All Fields])) OR (MMEJ
DNA Repair[All Fields])) OR (DNA Repair, MMEJ[All Fields])) OR (Repair, MMEJ DNA[All Fields])) OR
(Non-Homologous DNA End-Joining[All Fields])) OR (DNA End-Joining, Non-Homologous[All Fields]))
OR (End-Joining, Non-Homologous DNA[All Fields])) OR (Non Homologous DNA End Joining[All
Fields])) OR (Nonhomologous DNA End-Joining[All Fields])) OR (DNA End-Joining, Nonhomologous[All
Fields])) OR (Nonhomologous DNA End Joining[All Fields])) OR (NHEJ DNA Repair[All Fields])) OR
(DNA Repair, NHEJ[All Fields])) OR (Repair, NHEJ DNA[All Fields])))
```

Table S1. Newcastle-Ottawa Scale (NOS) Assessment of Included Studies

Study (first author, year)	Country	n	S1	S2	S3	S4	C1	C2	O1	O2	O3	Total (★)
Chen, 2019 [17]	Multicountry	350–429	★	★	★	★	☆	☆	★	★	★	7/9
Ricciuti, 2023 [18]	USA	1522+951	★	★	★	★	☆	☆	★	★	★	7/9
Ricciuti, 2020 [19]	USA	266	★	★	★	★	☆	☆	★	★	★	7/9
Sun, 2020 [20]	Multicountry	147+59	★	★	★	★	☆	☆	★	★	★	7/9
Zhang, 2021 [21]	China	429+240	★	★	★	★	☆	☆	★	★	★	7/9
Xiao, 2022 [22]	China	37	★	★	★	★	☆	☆	★	★	★	7/9
Averbuch, 2023 [23]	Israel	56	★	★	★	★	☆	☆	★	★	★	7/9
Gu, 2023 [24]	China	32	★	★	★	★	☆	☆	★	★	★	7/9
Kim, 2023 [25]	Korea	55	★	★	★	★	☆	☆	★	★	★	7/9
Xiong, 2021 [26]	Multicountry	429	★	★	★	★	☆	☆	★	★	★	7/9
Li, 2023 [27]	China	22	★	★	★	★	☆	☆	★	★	★	7/9
Wang, 2018 [28]	Multicountry	34	★	★	★	★	☆	☆	★	★	★	7/9
Olivares-Hernandez, 2022 [29]	Spain	73	★	★	★	★	☆	☆	★	★	★	7/9

S1: Representativeness of the DDR-mutated cohort (★ = truly/somewhat representative of real-world clinical patients receiving ICI therapy)

S2: Selection of the non-exposed cohort (★ = DDR wild-type patients drawn from the same institutional cohort)

S3: Ascertainment of exposure (★ = DDR mutation status determined by next-generation sequencing)

S4: Outcome of interest not present at start of study (★ = patients initiating ICI therapy at enrolment)

C1: Comparability — study controls for the most important confounder (★ = multivariable analysis adjusting for PD-L1 or disease stage)

C2: Comparability — study additionally controls for secondary confounders (★ = adjustment for TMB, histology, smoking, or co-mutations)

O1: Assessment of outcome (★ = outcomes ascertained from medical records per standard criteria)

O2: Follow-up long enough for outcomes to occur (★ = sufficient follow-up duration to capture OS/PFS/ORR endpoints)

O3: Adequacy of follow-up (★ = retrospective design with complete medical record data, negligible loss to follow-up)

All 13 included studies are retrospective cohort studies. No study reported multivariable analysis adjusting for key confounders (C1, C2 not awarded). Complete follow-up was achieved in all studies given the retrospective record-based design (O3 awarded for all). ★ = criterion met; ☆ = criterion not met. Score ≥7: high quality. Score 5–6: moderate quality.

Study or Subgroup	log[Hazard Ratio]	SE	Weight	Hazard Ratio IV, Random, 95% CI
Alejandro et al,.2022	−0.6931	1.5806	0.5%	0.50 [0.02, 11.08]
Averbuch et al,.2023	−0.5447	0.2025	15.7%	0.58 [0.39, 0.86]
Chen et al,.2019(c)	−0.755	0.2643	11.7%	0.47 [0.28, 0.79]
Chen et al,.2019(d)	−1.3471	0.5413	3.9%	0.26 [0.09, 0.75]
Li et al,.2023	−1.6094	0.8003	1.9%	0.20 [0.04, 0.96]
Ricciuti et al,.2020	−0.5447	0.1295	22.2%	0.58 [0.45, 0.75]
Ricciuti et al,.2023(a)	0.0296	0.1011	0.0%	1.03 [0.84, 1.26]
Ricciuti et al,.2023(b)	−0.1054	0.2939	10.2%	0.90 [0.51, 1.60]
Sun et al,.2020(a)	−0.3425	0.1589	19.4%	0.71 [0.52, 0.97]
Sun et al,.2020(b)	−1.0788	0.5314	4.0%	0.34 [0.12, 0.96]
Wang et al,.2018	−1.5606	0.6392	2.9%	0.21 [0.06, 0.74]
Xiao et al,.2022	−1.4697	0.4787	4.8%	0.23 [0.09, 0.59]
Zhang et al,.2021	0.0583	0.6633	2.7%	1.06 [0.29, 3.89]
**Total (95% CI)**			**100.0%**	**0.54 [0.43, 0.68]**

Heterogeneity: $Tau^2 = 0.04$; $Chi^2 = 16.55$, df = 11 (P = 0.12); $I^2 = 34\%$
Test for overall effect: Z = 5.39 (P < 0.00001)

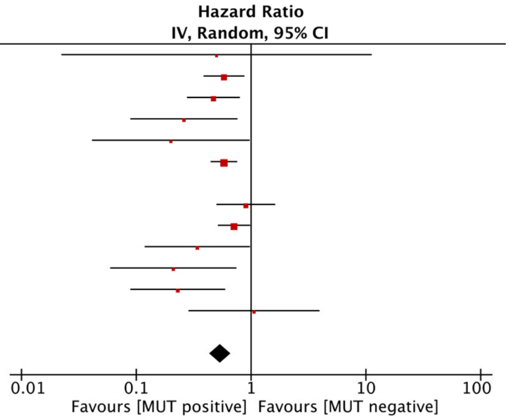

Figure S1. Sensitivity analysis for progression-free survival (PFS) in the overall population after exclusion of Ricciuti, 2023(a). Exclusion of this study reduced heterogeneity from 68% to 34%, indicating that the findings were robust and not driven by this individual study.

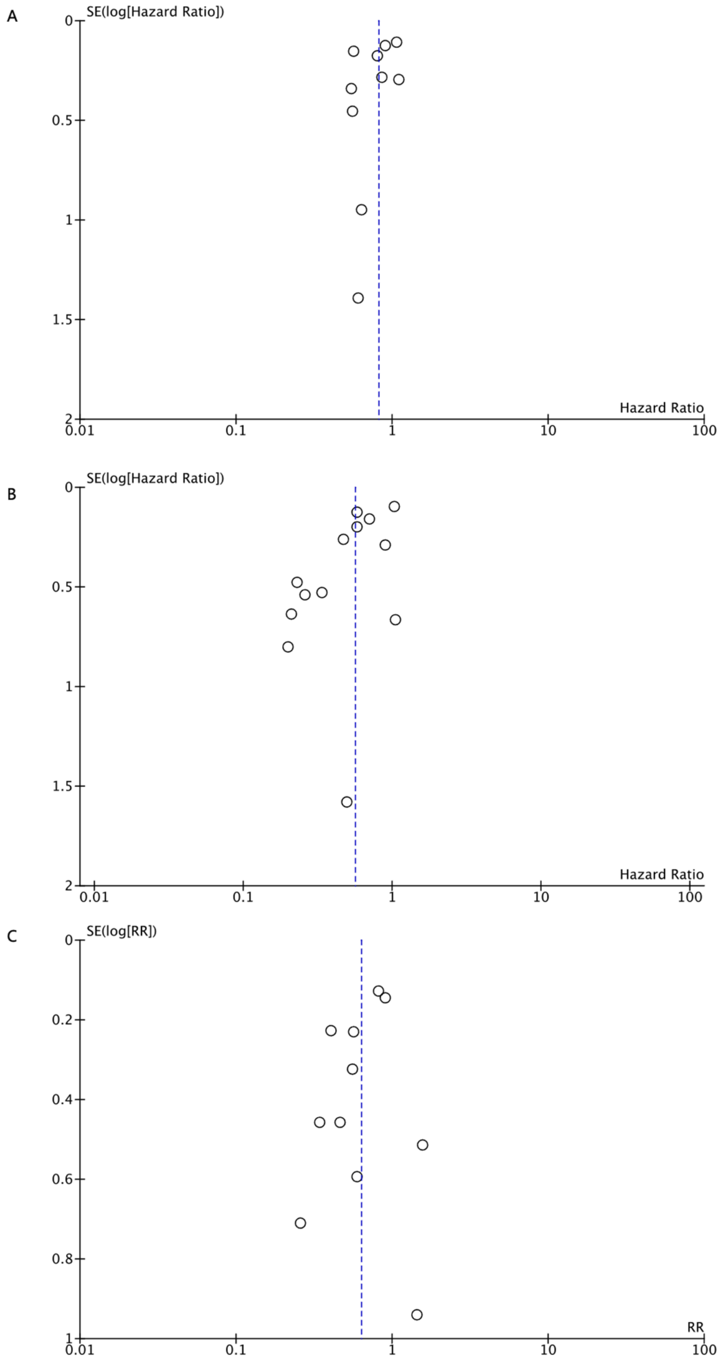

Figure S2. Funnel plots for overall survival (A), progression-free survival (B), and objective response rate (C) in the overall population. Each point represents an individual study. The plots appear approximately symmetric around the pooled effect estimates, with no substantial evidence of publication bias observed in any of the outcomes. No formal statistical tests for funnel plot asymmetry were performed due to the limited number of included studies.

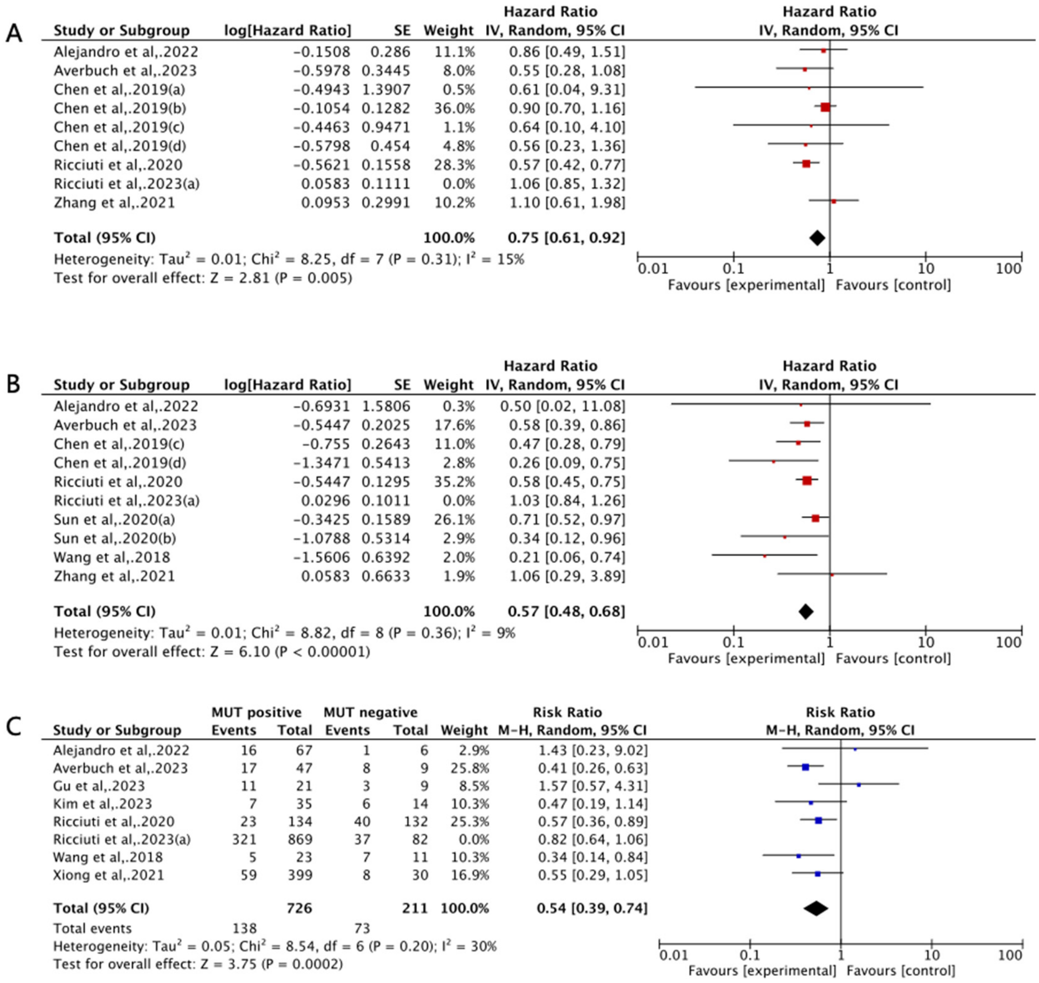

Figure S3. Sensitivity analyses for overall survival (A), progression-free survival (B), and objective response rate (C) in the ICI monotherapy subgroup after exclusion of Ricciuti, 2023(a). Exclusion of this study led to a reduction in heterogeneity across all outcomes: from 43% to 15% for OS, from 69% to 9% for PFS, and from 51% to 30% for ORR. The pooled hazard ratio for OS became statistically significant after exclusion, while the pooled estimates for PFS and ORR remained stable. These findings suggest that the results were robust and not driven by any single study.

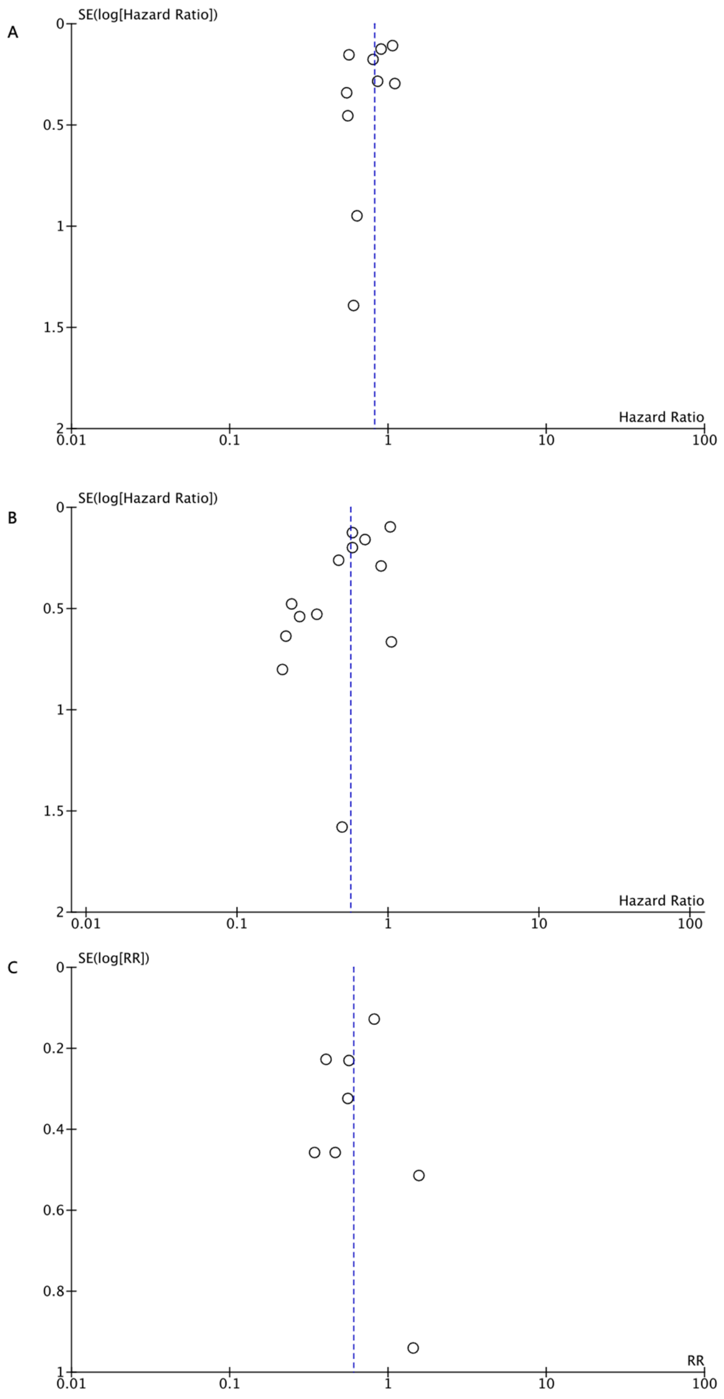

Figure S4. Funnel plots for overall survival (A), progression-free survival (B), and objective response rate (C) in the ICI monotherapy subgroup. Each point represents an individual study. All three plots demonstrate approximate symmetry around the pooled effect sizes, and no clear evidence of publication bias was observed. Due to the limited number of studies in each outcome, no formal statistical tests for funnel plot asymmetry were performed.

PRISMA 2020 Checklist

Association between mutations in DNA damage response genes and efficacy of immune checkpoint inhibitor therapy in non-small cell lung cancer: a systematic review and meta-analysis

Section/Topic	Item #	Checklist Item	Location in Manuscript
**TITLE**			
Title	1	Identify the report as a systematic review.	Title page: "…a systematic review and meta-analysis"
**ABSTRACT**			
Abstract	2	See the PRISMA 2020 for Abstracts checklist.	Structured Abstract: Background, Methods, Results, Conclusions; Keywords listed
**INTRODUCTION**			
Rationale	3	Describe the rationale for the review in the context of existing knowledge.	Introduction: limitations of PD-L1/TMB as predictive biomarkers; biological rationale for DDR mutations enhancing tumor immunogenicity via the cGAS-STING pathway; unmet need for reliable ICI predictors in NSCLC
Objectives	4	Provide an explicit statement of the objective(s) or question(s) the review addresses.	Introduction, final paragraph: states the aim to systematically evaluate the association between DDR gene mutations and ICI outcomes in NSCLC, with subgroup analysis by treatment regimen
**METHODS**			
Eligibility criteria	5	Specify the inclusion and exclusion criteria for the review and how studies were grouped for the syntheses.	Methods 2.1: five inclusion criteria — (1) observational cohort studies of NSCLC patients treated with ICIs; (2) comparative outcomes by DDR alteration status; (3) ICI monotherapy or combination therapy; (4) OS/PFS/ORR reported; (5) English language
Information sources	6	Specify all databases, registers, websites, organisations, reference lists and other sources searched. Specify the date when each source was last searched.	Methods 2.1: PubMed and Embase (controlled vocabulary + free-text terms); manual screening of reference lists. Search date: 30 May 2025
Search strategy	7	Present the full search strategies for all databases, including filters and limits used.	Appendix S1: full PubMed search strategy (MeSH + free-text terms for lung cancer, ICIs, and DNA repair/DDR pathways)
Selection process	8	Specify the methods used to decide whether a study met the inclusion criteria, including how many reviewers screened records, whether independently, and any automation tools used.	Methods 2.2: two reviewers (Li, Yiu) independently; disagreements resolved by discussion. Results 3.1 + Figure 1: 606 → 548 → 58 full texts → 13 included
Data collection process	9	Specify the methods used to collect data from reports, including how many reviewers, whether independently, and any automation tools used.	Methods 2.2: data independently extracted by two reviewers (Li, Yiu); disagreements resolved by discussion
Data items	10a	List and define all outcomes for which data were sought.	Methods 2.1 (criterion 4) + 2.3: OS (HR, 95% CI), PFS (HR, 95% CI), ORR (RR, 95% CI). Results: OS in 10 studies (n=5363), PFS in 13 studies (n=5033), ORR in 11 studies (n=3477)
	10b	List and define all other variables for which data were sought. Describe	Methods 2.2: first author, year, journal, design, total N, DDR-altered/wild-type N, stage, DDR genes, ICI type,

Section/Topic	Item #	Checklist Item	Location in Manuscript
		assumptions made about missing or unclear information.	treatment duration. DDR status extracted per original study definitions; cross-checked against the Wood Laboratory Human DNA Repair Genes resource (updated November 2025)
Study risk of bias assessment	11	Specify the methods used to assess risk of bias, including the tool(s) used and number/independence of reviewers.	Methods 2.2: Newcastle-Ottawa Scale (NOS) for cohort studies; assessed independently by two investigators; disagreements resolved by discussion (Supplementary Table S2)
Effect measures	12	Specify the effect measure(s) used for each outcome.	Methods 2.3: HR (95% CI) for OS and PFS; RR (95% CI) for ORR; two-sided $p<0.05$ considered significant
Synthesis methods	13a	Describe the processes used to decide which studies were eligible for each synthesis.	Methods 2.1 (criterion 4); Results 3.1: studies assigned to outcome pools by reported endpoints (OS n=10, PFS n=13, ORR n=11); subgroup pools split by treatment regimen
	13b	Describe methods to prepare data for synthesis (handling of missing statistics, data conversions).	Not separately described as a data-conversion step. DDR status extracted per original study definitions without reclassification (Methods 2.2)
	13c	Describe methods used to tabulate or visually display results.	Table 1: characteristics of all 13 included studies. Figures 2–4: forest plots for OS, PFS, ORR (overall and by treatment regimen)
	13d	Describe methods used to synthesize results; if meta-analysis performed, describe model(s), heterogeneity method(s), and software.	Methods 2.3: random-effects model; χ^2 test and I^2 statistic ($p<0.10$ = significant heterogeneity; $I^2>50\%$ = substantial); Review Manager (RevMan) version 5.4
	13e	Describe methods used to explore possible causes of heterogeneity.	Methods 2.3 + Results: pre-specified subgroup analysis by treatment regimen (ICI monotherapy vs. ICI + chemotherapy)
	13f	Describe any sensitivity analyses conducted.	Results: leave-one-out sensitivity analysis by sequential exclusion of individual studies (Supplementary Figure 1, overall PFS; Supplementary Figure 3A-C, ICI monotherapy subgroup)
Reporting bias assessment	14	Describe methods used to assess risk of bias due to missing results.	Methods 2.3: visual inspection of funnel plots. No formal statistical test performed due to the limited number of included studies
Certainty assessment	15	Describe methods used to assess certainty in the body of evidence.	Not performed. A formal GRADE assessment was not conducted, given the retrospective observational design of all included studies; this is noted as a limitation in the Discussion
**RESULTS**			
Study selection	16a	Describe the results of the search and selection process, ideally with a flow diagram.	Results 3.1: 606 records identified → 58 duplicates removed → 548 screened → 490 excluded → 58 full texts assessed → 45 excluded → 13 included. Figure 1: PRISMA flow diagram
	16b	Cite studies that might appear eligible but were excluded, and explain why.	Results 3.1: excluded full-text studies described by category (reviews/case reports; ineligible design; inappropriate population; outcomes not reported). Individual citations not separately listed
Study characteristics	17	Cite each included study and present its characteristics.	Table 1: all 13 included studies (refs 17–29) with author, year, country, design, DDR genes, patient numbers, ICI regimen, stage, outcomes reported

Section/Topic	Item #	Checklist Item	Location in Manuscript
Risk of bias in studies	18	Present risk-of-bias assessments for each included study.	Supplementary Table S2: NOS assessment for all 13 included studies; all studies scored 7/9, reflecting generally high methodological quality with the main limitation being lack of multivariable confounder adjustment
Results of individual studies	19	For all outcomes, present summary statistics and effect estimates with precision for each study.	Figures 2–4: forest plots showing per-study HR or RR with 95% CI for OS, PFS, and ORR, overall and by treatment-regimen subgroup
Results of syntheses	20a	Briefly summarise the characteristics and risk of bias among studies contributing to each synthesis.	Results: each analysis subsection reports contributing study counts, patient numbers, and heterogeneity statistics
	20b	Present results of all statistical syntheses, including summary estimates, precision, and heterogeneity measures.	Results, "Overall Impact…": OS HR=0.82 (95% CI 0.68–0.98, I^2=36%); PFS HR=0.56 (95% CI 0.43–0.74, I^2=68%); ORR RR=0.63 (95% CI 0.49–0.82, I^2=50%). Subgroup results in "Subgroup Analyses by Treatment Regimen"; Figures 2–4
	20c	Present results of investigations into possible causes of heterogeneity.	Results, "Subgroup Analyses by Treatment Regimen": treatment regimen identified as a source of heterogeneity. Sensitivity analysis identified Ricciuti 2023(a) as a major contributor (Supplementary Figures 1 and 3A-C)
	20d	Present results of sensitivity analyses.	Results: excluding Ricciuti 2023(a) reduced overall PFS I^2 from 68% to 34% (HR stable, Supplementary Figure 1); in the monotherapy subgroup, heterogeneity fell for OS (43%→15%), PFS (69%→9%), and ORR (51%→30%), with the OS estimate becoming statistically significant (Supplementary Figure 3A-C)
Reporting biases	21	Present risk-of-bias-due-to-missing-results assessments for each synthesis.	Overall population: funnel plots approximately symmetric (Supplementary Figure 2A-C). ICI monotherapy subgroup: funnel plots approximately symmetric (Supplementary Figure 4A-C). No formal Egger's/Begg's test performed due to the limited number of studies
Certainty of evidence	22	Present certainty assessments for each outcome.	Not performed (see Item 15); noted as a limitation in the Discussion
**DISCUSSION**			
Discussion	23a	Provide a general interpretation of results in the context of other evidence.	Discussion, opening paragraphs: results interpreted against prior retrospective cohorts and biological plausibility (cGAS-STING pathway); treatment-context dependence highlighted as the principal novel finding
	23b	Discuss limitations of the evidence included.	Discussion, Limitations: all included studies retrospective; PD-L1, TMB, smoking history, and STK11/KEAP1 co-mutation data inconsistently available; no uniform non-ICI comparator; heterogeneous DDR definitions across studies
	23c	Discuss limitations of the review process.	Discussion, Limitations: limited number of eligible studies for regimen-specific subgroup analyses; prospective validation needed; multivariable adjustment not feasible from published data
	23d	Discuss implications for practice, policy, and future research.	Discussion: recommends pathway-resolved biomarker models and biomarker-enriched trials of ICI monotherapy or DDR-targeting combinations;

Section/Topic	Item #	Checklist Item	Location in Manuscript
			Supplementary Table S1 summarises the current clinical trial landscape
**OTHER INFORMATION**			
Registration and protocol	24a	Provide registration information, or state that the review was not registered.	This review was not prospectively registered (e.g., in PROSPERO)
	24b	Indicate where the protocol can be accessed, or state that a protocol was not prepared.	No review protocol was prepared prior to conducting this review
	24c	Describe and explain any amendments to information provided at registration or in the protocol.	Not applicable — no registration or protocol
Support	25	Describe sources of financial or non-financial support, and the role of funders.	Funding statement: Shenzhen Medical Research Fund (D2402020); National Natural Science Foundation of China (82573463); Shenzhen-Hong Kong Cooperation Zone for Technology and Innovation (HZQSWS-KCCYB-2024055); Shenzhen Science and Technology Program (ZDSYS20210623091811035). Funders had no role in study design, conduct, analysis, or the decision to publish
Competing interests	26	Declare any competing interests of review authors.	Conflicts of Interest: "The authors declare no conflicts of interest."
Availability of data, code and other materials	27	Report which data, code, and materials are publicly available and where.	Data Availability Statement: "No new data were created or analyzed in this study. Data sharing is not applicable to this article."

From: Page MJ et al. The PRISMA 2020 statement: an updated guideline for reporting systematic reviews. BMJ 2021;372:n71. doi: 10.1136/bmj.n71. This checklist is licensed under CC BY 4.0.
